# Evaluation of FRP Confinement Models for Substandard Rectangular RC Columns Based on Full-Scale Reversed Cyclic Lateral Loading Tests in Strong and Weak Directions

**DOI:** 10.3390/polym8090323

**Published:** 2016-09-02

**Authors:** Hamid Farrokh Ghatte, Mustafa Comert, Cem Demir, Alper Ilki

**Affiliations:** 1 Technical and Vocational University (TVU), Brazil St., No. 4, Tehran 1435761137, Iran; 2 Istanbul Technical University, Ayazaga, Sariyer, Istanbul 34469, Turkey; mcomert@itu.edu.tr (M.C.); demirce@itu.edu.tr (C.D.); ailki@itu.edu.tr (A.I.)

**Keywords:** column, confinement, FRP, reinforced concrete, retrofitting, seismic, substandard

## Abstract

Although many theoretical and experimental studies are available on external confinement of columns using fiber-reinforced polymer (FRP) jackets, as well as numerous models proposed for the axial stress-axial strain relation of concrete confined with FRP jackets, they have not been validated with a sufficient amount and variety of experimental data obtained through full-scale tests of reinforced concrete (RC) columns with different geometrical and mechanical characteristics. Particularly, no systematical experimental data have been presented on full-scale rectangular substandard RC columns subjected to reversed cyclic lateral loads along either their strong or weak axes. In this study, firstly, test results of five full-scale rectangular substandard RC columns with a cross-sectional aspect ratio of two (300 mm × 600 mm) are briefly summarized. The columns were tested under constant axial load and reversed cyclic lateral loads along their strong or weak axes before and after retrofitting with external FRP jackets. In the second stage, inelastic lateral force-displacement relationships of the columns are obtained analytically, making use of the plastic hinge assumption and different FRP confinement models available in the literature. Finally, the analytical findings are compared with the test results for both strong and weak directions of the columns. Comparisons showed that use of different models for the stress-strain relationship of FRP-confined concrete can yield significantly non-conservative or too conservative retrofit designs, particularly in terms of deformation capacity.

## 1. Introduction

A significant amount of the existing RC buildings, particularly in developing countries, has typical deficiencies, like low quality of concrete, poor details of reinforcement, use of plain bars, lack of adequate transverse reinforcement for shear effects and inadequate confinement reinforcement at the potential plastic hinging regions. Thus, many existing substandard RC buildings are in urgent need of seismic retrofitting, particularly in terms of ductility, to achieve an acceptable performance during seismic actions. The fiber-reinforced polymer (FRP) composites have been widely used in reinforced concrete (RC) structures for repair and retrofit purposes in recent years. Many studies have demonstrated the efficiency of FRPs for the repair and retrofit of typical RC members [1,2,3,4,5,6,7,8,9,10,11,12,13,14,15,16,17], and numerous models have been proposed for the axial stress-axial strain relationship of FRP-confined concrete [18,19,20,21,22,23,24,25,26,27,28,29]. Although external FRP jacketing is a promising and feasible retrofit method for the enhancement of the ductility of substandard columns, a limited number of studies has been reported on the efficiency of FRP retrofitting for RC columns [30,31,32,33,34,35,36,37,38,39,40,41,42,43,44,45]. For instance, the extensive column test database summarized in [30] mentions 203 FRP confined column tests, whereas the number of column tests without FRP wrapping hits a large number over 2500. According to the best knowledge of the authors, no experimental data were reported on full-scale substandard rectangular FRP retrofitted RC columns tested along their weak axes. In addition, no comprehensive evaluation of analytical predictions made using different models of the stress-strain relationship of FRP-confined concrete was reported. Therefore, in this study, five cantilever full-scale substandard RC columns with a cross-sectional aspect ratio of two (600 mm × 300 mm) are tested under constant axial load and reversed cyclic lateral loads in strong or weak directions. The details of the tested columns are realistic and represent the features of existing substandard columns. While two of the specimens were tested as the reference specimens (one tested along the strong, the other along the weak axis), the others were tested after jacketing externally with one or two plies of carbon fiber-reinforced polymer (CFRP) composites at the potential plastic hinging region. Initially, the damage evolution on reference and retrofitted RC columns and observed behavior are summarized. Then, the strength and deformability characteristics of the reference and retrofitted specimens are compared. Finally, the behavior of the tested columns is analyzed through a nonlinear inelastic approach making use of the plastic hinge concept [5,43,46,47] and several analytical models that have been proposed for the stress-strain relationship of FRP-confined concrete under compression [20,22,24,27]. The findings based on the comparison of experimental and analytical results in terms of lateral load-drift ratio relationships showed that the use of different available models could yield significantly different nonlinear behavior characteristics of retrofitted RC columns under lateral loads simulating seismic actions. Naturally, this can cause remarkably non-conservative or too conservative estimation of the seismic performance of FRP-retrofitted RC columns. Based on this finding, an available FRP stress-strain model of Ilki et al. [22] is modified to capture the inelastic deformation capacities of substandard FRP retrofitted columns realistically. Additionally, the experimental results are compared with the predictions of the American Concrete Institute (ACI) 440.2R [48] approach in terms of the strength and ductility of FRP-confined RC columns.

## 2. Experimental Study

**Figure 1:**
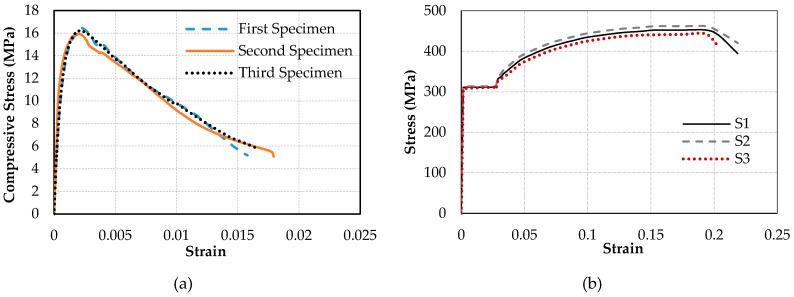
(**a**) Concrete stress-strain relationships; (**b**) rebar stress-strain relationships. S1, S2 and S3 are specimens with 14 mm-diameter longitudinal bars.

**Figure 2:**
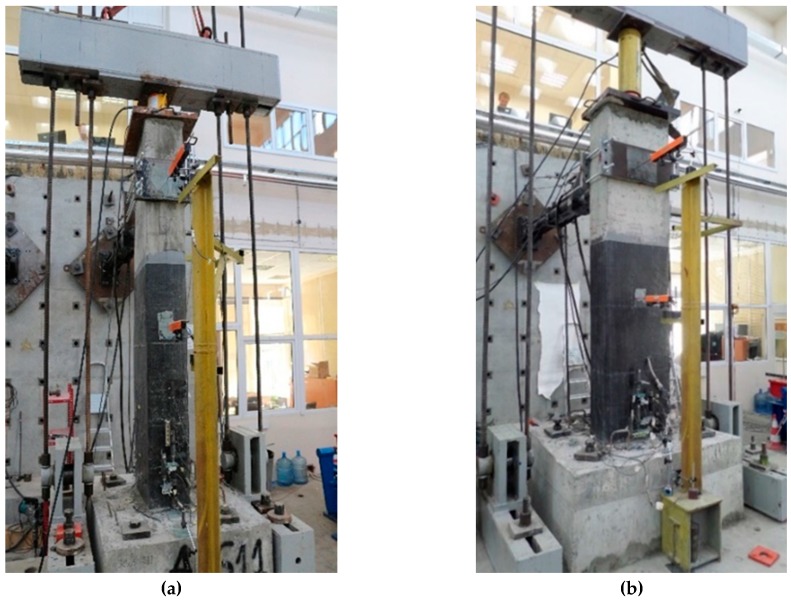
Test setup for (**a**) the first and (**b**) the second group of specimens.

**Figure 3:**
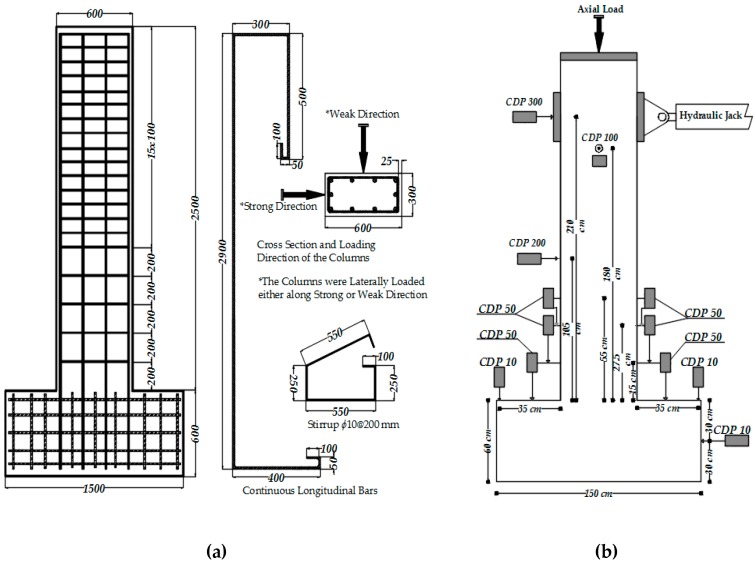
(**a**) Reinforcement scheme (dimensions in mm); (**b**) test setup.

Five substandard full-scale rectangular cantilever RC columns with a cross-sectional aspect ratio of two (600 mm × 300 mm) were tested under constant axial load and reversed cyclic lateral load. The substandard features of the tested columns were low quality of concrete, plain reinforcing bars and large spacing and low volumetric ratio of transverse reinforcement. The specimens were cast in two parts, foundations (1500 mm × 1000 mm × 600 mm) and columns, for which the average standard cylinder compressive strength of concrete at 180 days (*f’*_c_) was 30 MPa and 16 MPa, respectively. The stress-strain relationships of concrete used for the construction of the columns are presented in Figure 1a. Plain bars were used for the reinforcement of the columns with average yield strengths (*f*_y_) of 310 and 330 MPa, for longitudinal and transverse bars, respectively. The average mechanical characteristics of 14 mm-diameter longitudinal and 10 mm-diameter transverse plain bars are given in Table 1, where *f*_y_, *f*_max_ and *f*_u_ are yield, maximum and ultimate tensile stresses and ε_y_, ε_max_, and ε_u_ are the tensile strains corresponding to *f*_y_, *f*_max_ and *f*_u_, respectively. The stress-strain relationships for 14 mm-diameter longitudinal bars (S1, S2 and S3 specimens) are presented in Figure 1b. Three of the columns were seismically retrofitted by using CFRP sheets externally wrapped around the columns in the transverse direction. The mechanical and geometrical properties of the carbon fiber sheets (as indicated by the manufacturer) are presented in Table 2. The columns were subjected to reversed cyclic lateral displacements while they were subjected to a constant axial load that corresponded to 35% of the axial capacities of the columns (excluding axial force capacity of the longitudinal bars). Therefore, the constant axial load on the columns was 1000 kN. It should be noted that, in the case of substandard buildings, the column axial load levels are considerably high, which is expected to significantly decrease the deformation capacity (or ductility) of the columns during seismic actions. As also indicated by Tapan et al. [49], this is mainly due to lower concrete quality and/or smaller dimensions of vertical structural members compared to concrete quality and column cross-section dimensions considered during the design phase. On the other hand, this high column axial load level is expected to increase the efficiency of FRP confinement, which requires lateral expansion of concrete for the activation of the confinement mechanism. The specimens were classified into two categories; the first group included three specimens that have been tested in the strong direction (specimens REF-35, RET-35-N1 and RET-35-N2), while the second group included two specimens that have been tested in the weak direction (specimens REF-W-35 and RET-W-35-N2, W for weak). For both groups, one specimen was the un-retrofitted (reference, indicated as REF) column, and the remaining one(s) were jacketed externally (retrofitted, indicated as RET) either with one (N1) or two (N2) layers of CFRP sheets. The main characteristics of the specimens, such as the name, loading direction and retrofit method, are presented in Table 3. All specimens were tested using the setup shown in Figure 2. The vertical distance between the top of the foundation and the center of the actuator was 2100 mm. At different stages of testing, damage observations, such as the locations and extents of cracks, cover spalling and crushing of concrete, were recorded. The test setup, geometry of the columns and the reinforcement details are shown in Figure 3.

### 2.1. Description of the Specimens

The full-scale columns with the cross-section dimensions of 300 mm × 600 mm were intentionally constructed with low-quality concrete and plain reinforcing bars for representing the substandard columns. The geometric ratio of the longitudinal 14 mm-diameter steel reinforcement (ten 14-mm bars) was 0.0085. The clear cover was 25 mm over the transverse reinforcement. All specimens were constructed by using 90° hooked 10 mm-diameter stirrups with a spacing of 200 mm. The transverse steel reinforcement ratios (*A*_sh_/*sb*_k_) were 0.0026 and 0.0013 for the R35 and RW35 series, respectively, where *A*_sh_ is the total cross-sectional area of transverse reinforcement with spacing s and perpendicular to core dimension *b*_k_ in the cross-section (Figure 3a).

The columns REF-35 and REF-W-35 were the reference specimens in their series. These columns were designed to fail in flexure and exhibit low ductility due to premature crushing of concrete and buckling of longitudinal bars. The specimens RET-35-N1, RET-35-N2 and RET-W-35-N2 were retrofitted with external CFRP jackets with fibers aligned in the transverse direction. All corners of the rectangular cross-sections of these columns were rounded to a 30-mm radius before CFRP jacketing to satisfy the requirement of the Turkish Seismic Design Code (TSDC 2007) [50]. The specimen RET-35-N1 was jacketed with one layer of CFRP sheet. The specimens RET-35-N2 and RET-W-35-N2 were retrofitted by external jackets of two layers of CFRP sheets. The geometric ratios of CFRP confinement reinforcement (ρ_CFRP_) were 0.00166 and 0.00332 for columns jacketed with one and two layers of CFRP sheets, respectively. For avoiding failure due to the loss of the bond of CFRP sheets, an overlap of a 200-mm length was formed at the end of the CFRP jackets.

### 2.2. Test Setup and Loading Pattern

**Figure 4:**
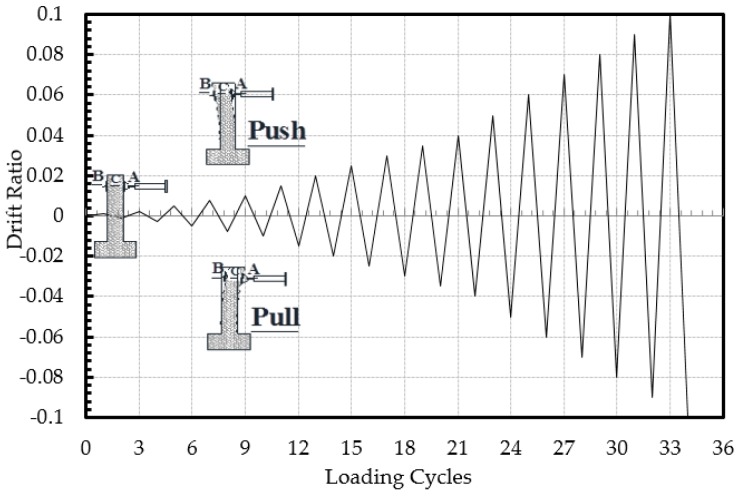
Loading protocol used for the cyclic tests.

The specimens were subjected to combined action of axial load and reversed cyclic lateral load. As seen in Figure 2, the axial load was applied by using a manually-controlled hydraulic jack placed between the top face of the column and a rigid steel beam, which was connected to the strong floor with four PC bars. The axial load was measured using a 1500-kN capacity load cell. A 250-kN capacity servo-controlled hydraulic actuator, connected to the columns at a 2100-mm height from the top of the foundation, was used to apply the lateral load in the form of reversed displacement cycles. The reversed cyclic lateral loading was applied with increasing top displacements in pushing and pulling directions that corresponded to ±0.001, ±0.0025, ±0.005, ±0.0075, ±0.01, ±0.015, ±0.02, ±0.025, ±0.03, ±0.035, ±0.04, ±0.05 ±0.06, ±0.07 and ±0.08 drift ratios until failure (loss of lateral and/or axial load capacities). The drift ratio is the ratio of top horizontal displacement of the column (at the center of the actuator) to the height of the column (from the top of the foundation to the center of the actuator). The loading protocol applied according to the targeted drift ratios is shown in Figure 4. A number of horizontally- and vertically-oriented displacement transducers (LVDTs) were used to measure the absolute and relative displacements of the specimens (Figure 3b). The measured displacements were mainly used to calculate top drift ratios and average curvatures over different gauge lengths of the columns above the column-foundation interface, where the bending effects were maximum. For all of the specimens, the translation and rotation of the foundation, as well as out-of-plane displacement of the column were also recorded during the test to prove that they were small enough and negligible. The measurements of sensors were collected and stored by a data logger.

### 2.3. Failure Patterns and Performances of Tested Columns

**Figure 5:**
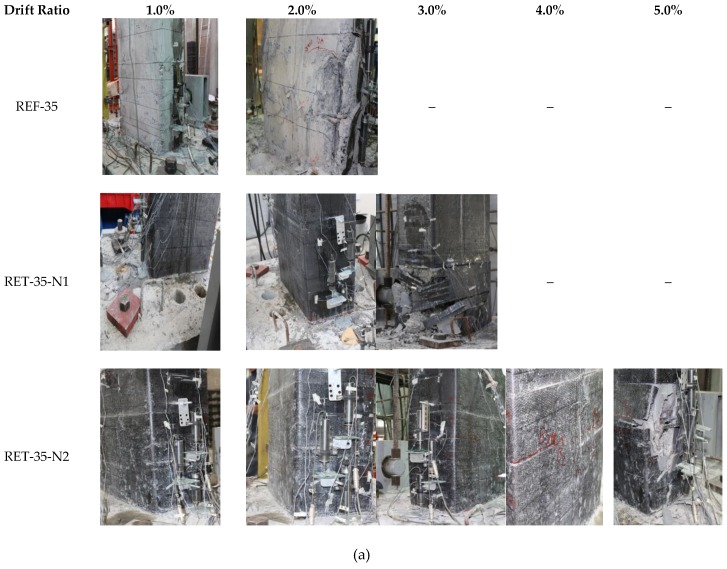

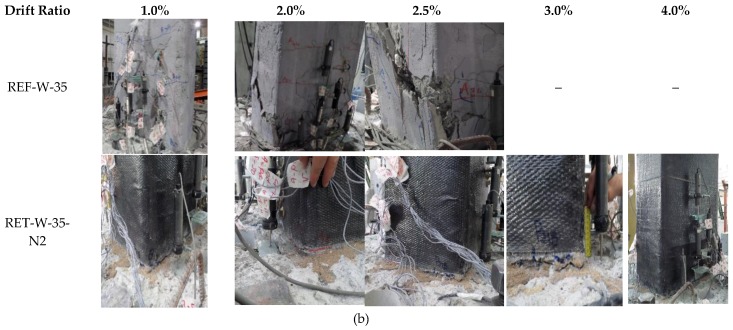
(**a**) Damage evolution of the R35 series; (**b**) damage evolution of the RW35 series.

Although the lateral loading was continued until the development of significant damage and loss of strength, in this study, the ultimate condition is assumed to be at the displacement, which corresponds to 15% loss in lateral strength or sudden rupture of the FRP jacket before a 15% strength loss takes place. The 15% strength loss definition for ultimate condition is a widely-accepted assumption among other percentages, such as 20% or 25%. Since the behavior of sub-standard reinforced concrete members is rather brittle due to premature crushing of concrete or buckling of longitudinal bars, a percentage of 15% is selected here. The evolution of damage can be seen in Figure 5a for the R35 series and in Figure 5b for the RW35 series columns. In addition, lateral load and drift ratio values corresponding to cracking of concrete (*P*_cr_ and *DR*_cr_), yielding of the longitudinal bars (*P*_y_ and *DR*_y_), peak load (*P*_max_ and *DR*_max_) and ultimate condition (*P*_u_ and *DR*_u_) are summarized in Table 4.

#### 2.3.1. R35 Series

**Figure 6:**
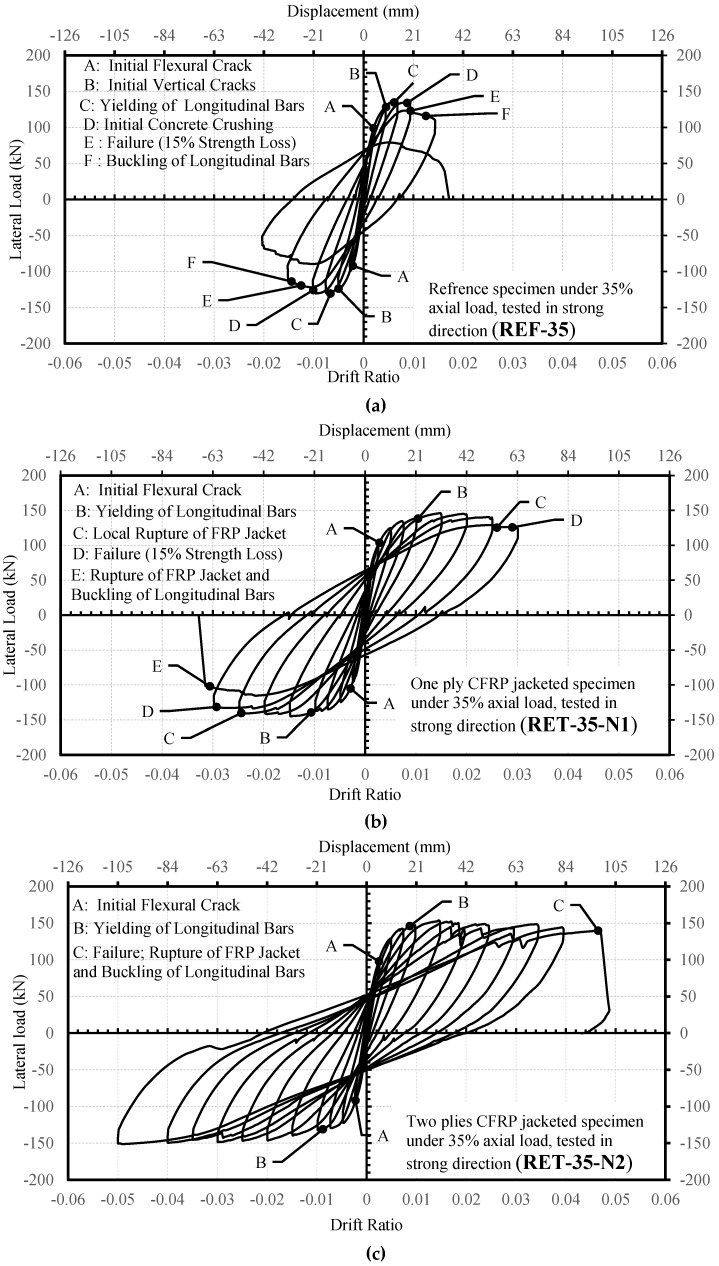
Lateral load-drift ratio relationships for the R35 series: (**a**) REF-35, (**b**) RET-35-N1; and (**c**) RET-35-N2.

The lateral force-drift ratio relationships of the specimens are presented in Figure 6. The reference specimen REF-35 reached the peak strength of 136.9 kN at a lateral displacement of 14.3 mm (0.7% drift ratio). Vertical cracks were observed in the compression zone at drift ratios around 0.5% because of the high axial load level. The ultimate displacement was 30.2 mm (when 15% loss was measured in lateral load capacity) corresponding to a drift ratio of 1.5%. Finally, all lateral load capacity was lost suddenly in a brittle manner because of the buckling of longitudinal bars in compression and the crushing of concrete at around a 2% drift ratio. The specimen RET-35-N1 reached the peak strength of 145 kN at a lateral displacement of 31.1 mm, corresponding to a drift ratio of 1.5%. The ultimate displacement was 62.3 mm at the drift ratio of 3%. Finally, the test was stopped at around a 3.2% drift ratio because of the rupture of the CFRP jacket in the plastic hinge zone and buckling of longitudinal column bars. Nevertheless, a remarkable enhancement was obtained in terms of ductility with respect to the reference column. The specimen RET-35-N2 reached the peak strength of 152.6 kN at a lateral displacement of 30.8 mm, corresponding to a drift ratio of 1.5%. The ultimate displacement was 105 mm (approximately corresponding to 5% drift ratio) at which the lateral load capacity was almost totally lost due to the rupture of the CFRP jacket followed by the buckling of longitudinal bars. The ductility of this column retrofitted with a jacket of two layers of CFRP sheets was remarkably higher than the reference column (REF-35) and the column retrofitted with a jacket of one layer of CFRP sheet (RET-35-N1).

#### 2.3.2. RW35 Series

**Figure 7:**
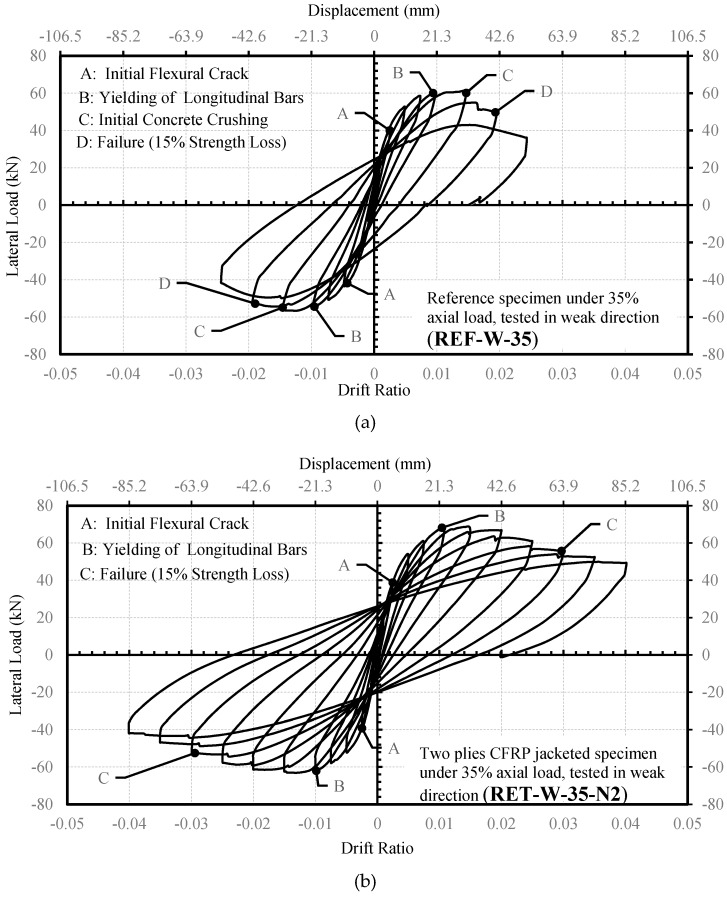
Lateral load-drift ratio relationships for the RW35 series: (**a**) REF-W-35; and (**b**) RET-W-35-N2.

The lateral force-drift ratio relationships of the specimens are given in Figure 7. The specimen REF-W-35 reached a peak lateral load of 60.7 kN at a lateral displacement of 31.2 mm (1.5% drift ratio). For this column, the ultimate displacement was 41.7 mm (2% drift ratio). The specimen RET-W-35-N2 reached the peak strength of 68.9 kN at a lateral displacement of 31.4 mm (1.5% drift ratio), and the displacement corresponding to the ultimate condition (ultimate displacement) was 63.9 mm (3% drift ratio). It should be noted that no sudden failure was observed until a 4% drift ratio, where the test was stopped intentionally due to gradual loss of lateral strength. At 4% drift, debonding between the CFRP jacket and concrete was observed due to vertical cracks and crushing of concrete in compression, and the loss in the lateral load capacity was approximately 30%. The significant increase in drift capacity shows that FRP confinement is effective for enhancing the ductility of the columns (with a cross-sectional aspect ratio of two) subjected to lateral loads along their weak axes, as well.

## 3. Investigated Stress-Strain Models for FRP Confined Concrete

**Figure 8:**
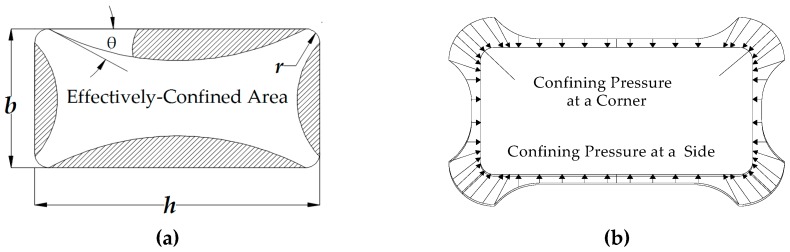
(**a**) Effectively-confined area; (**b**) Confining pressure at a corner and a side (modified from [51]).

Many models have been proposed by different researchers for the stress-strain behavior of concrete externally confined uniformly with FRPs [18,19,20,21,22,23,24,25,26,27,28,29]. Most of these models are empirical and based on compression tests of small-sized cylinders. On the other hand, as seen in Figure 8 [51], under concentric compression loading, the confinement stresses are not uniformly distributed when the cross-section of the structural member is not circular. Additionally, when the column is subjected to combined axial load and bending effects (eccentric loading), the confinement pressure also exhibits a non-uniform distribution [52]. As also indicated by Wu and Jiang [53] and Wu [54], very few stress-strain models have been developed for the stress-strain behavior of FRP-confined concrete subjected to eccentric loading conditions. Additionally, the existing results and modelling approaches contradict each other [54]. Moreover, the load path dependence of the stress-strain model (columns loaded with constant eccentricity and increasing axial load or columns loaded with constant axial load and increasing eccentricity) is another issue that needs to be investigated. Considering that the eccentric loading issue is still a topic that needs to be further studied for low-strength concrete and rectangular cross-sections, in this study, confined concrete models obtained from concentric loading are utilized and compared. This is also in parallel with the design approaches followed in the current design documents for transverse steel reinforcement (stirrups or hoops) or FRP-confined reinforced concrete columns subjected to combined loading (axial load and bending moment effects). Obviously, the efficiency of confinement is significantly reduced when confinement stresses are not uniformly distributed [55]. Furthermore, the required confinement reinforcement for a ductile behavior increases when the section dimensions or cross-sectional aspect ratios increase.

For FRP-confined non-circular sections, for which confinement stresses are not uniform, the number of stress-strain models proposed up to date is limited. In addition, experimental data on relatively larger scale non-circular columns with actual reinforcing bars are scarce. Therefore, validation of the proposed models for actual size FRP-retrofitted RC columns with longitudinal and transverse steel reinforcing bars is vitally important. Consequently, in this study, different axial stress-axial strain models proposed for FRP-confined concrete are used for nonlinear structural analysis of the tested full-scale FRP retrofitted RC columns to investigate their suitability for the prediction of the behavior under axial and reversed cyclic lateral loads representing seismic actions. The stress-strain models investigated in this study are the ones proposed by Lam and Teng [20], Ilki et al. [22], Youssef et al. [24] and Hany et al. [27]. These models are applicable to rectangular cross-sections, and as seen in Table 5, they are based on uniaxial compression tests of low to medium strength concrete prisms with cross-sectional aspect ratios varying between one and 1.5. The main characteristics of the tested columns (such as concrete strength, cross-section dimensions, retrofit scheme and material mechanical properties) are within the scope of the investigated models, and they are applicable without any apparent dependency on any of these parameters. In general, the rupture strain of fiber-reinforced polymer jackets is lower than the ultimate tensile strain of the component fibers [56], and among the investigated models, except Youssef et al. [24], all of the models consider an efficiency factor (*k*_ε_) for FRPs. Table 6 summarizes the expressions proposed by these models for predicting the FRP-confined concrete ultimate strength (*f*_cc_) and ultimate strain values (ε_cc_). Further details about the models can be found elsewhere [20,22,24,27]. In Table 5, *f*_co_ is the unconfined concrete compressive strength; ε_co_ is the axial strain corresponding to unconfined concrete strength; (*h/b*) is the cross-section aspect ratio (where *h* is the longer and *b* is the shorter side of the rectangular cross-section); *f*_l_ is the lateral confinement pressure; *f’*_l_ is the effective lateral confinement pressure; *k*_s1_ and *k*_s2_ are the shape factors for compressive strength and ultimate axial strain, respectively; ε_h,rup_ (*k*_ε_ε_fu_) is the actual hoop rupture strain, and ε_fu_ is the ultimate tensile strain of the FRP jacket; *f*_ju_ is the tensile strength, and *E*_j_ is the tensile modulus of the FRP jacket in the hoop direction, respectively; *A*_e_ is the effectively-confined cross-section area (Figure 8a); *A*_c_ is the gross cross-section area, and LTF is the loading type factor to be taken into account as one for monotonic and two for cyclic loading.

**Figure 9:**
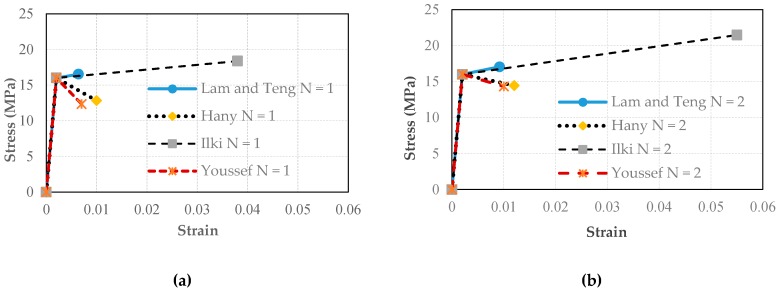
Axial stress-strain relationships for CFRP-jacketed columns obtained from model predictions: (**a**) one ply; (**b**) two plies.

In order to compare the predictions of the above-mentioned models for the compressive behavior of the tested column cross-sections, axial stress-strain diagrams are plotted as seen in Figure 9. For this purpose, the stress-strain diagrams are defined by means of bilinear curves, which consist of three data points: origin, the point corresponding to unconfined concrete strength and the strain corresponding to it (*f*_co_, ε_co_) and FRP-confined concrete strength and ultimate axial strain (*f*_cc_, ε_cc_). Here, the unconfined concrete strength is assumed as the value obtained from standard cylinder tests (16 MPa) and the corresponding strain (ε_co_) as 0.002. For each model, the FRP-confined concrete strength (*f*_cc_) and ultimate axial strain (ε_cc_) values are obtained by using the equations provided in Table 6, and their ratios to *f*_co_ and ε_co_ are tabulated together with the effective confinement pressure (*f’*_l_) values, as seen in Table 7. The bilinear curve simplification has only marginal influence on the overall behavior, particularly when the main focus is on ductility. The contribution of internal transverse steel reinforcement to the confinement is neglected because of large spacing and the low volumetric ratio of this reinforcement. In addition, since no transverse cross-ties were used, the efficiency of these bars is very low. Comparison of the obtained simplified axial stress-strain relationships points to an interesting and significant difference among different FRP-confined concrete models. For both one and two plies of FRP jacket and for both the R35 and RW35 series, the equations of Ilki et al. [22] gives significantly higher ultimate axial strain values; while the model based on the equations of Lam and Teng [20] gives a similar slope for the part of the stress-strain relationship between the unconfined and confined concrete strengths; like the model proposed by Ilki et al. [22], the ultimate strain and ultimate strength are significantly less (Figure 9). In contrast to the models based on the equations of Ilki et al. [22] and Lam and Teng [20], the models based on the equations proposed by Youssef et al. [24] and Hany et al. [27] give a negative slope for the post-peak part of the FRP-confined concrete stress-strain relationship. It should be noted that the slope of the second branch is mainly related to the stiffness of the confining jacket, thus the confinement ratio (ratio of the confinement pressure to concrete strength). In the second branch (following the ascending first branch) the FRP confinement is activated as concrete dilates laterally as a result of concrete crushing. If the confinement ratio (or the stiffness of the jacket) is low, the jacket cannot restrain the lateral dilation of concrete, which causes the descending branch.

## 4. Comparison of Analytical and Experimental Load-Displacement Relationships

**Figure 10:**
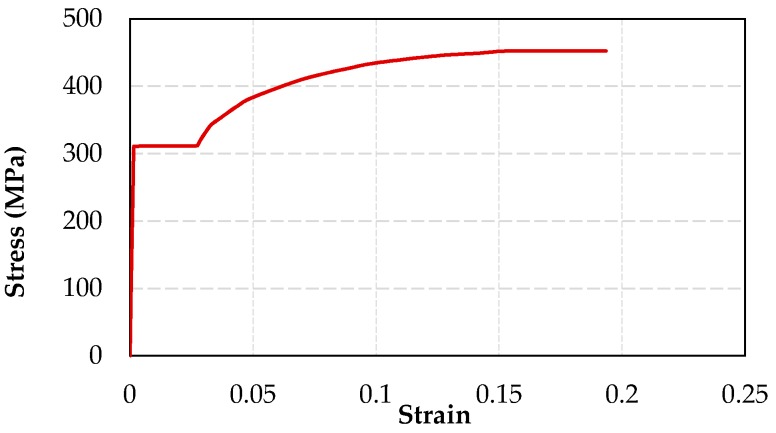
Axial stress-strain relationship assumed for steel bars.

For obtaining the analytical load-displacement relationships of the tested FRP-jacketed columns, the moment-curvature relationships for the critical cross-sections (potential plastic hinging regions) of the columns are required. In this study, the fiber analysis approach was used, for which the confined concrete behavior was considered by using the Lam and Teng [20], Ilki et al. [22], Youssef et al. [24] or Hany et al. [27] FRP-confined concrete models. The contribution of the longitudinal steel reinforcement was represented by using an elasto-plastic stress-strain curve with strain hardening as seen in Figure 10. The idealized experimental stress-strain curve for steel bars was defined by using the results of uniaxial steel tension tests.

The final stage of constructing inelastic load-displacement relationships of the columns was the nonlinear analysis of the columns making use of plastic hinge theory. In this stage, the assumption made for the plastic hinge length of the column is vitally important, since this affects the displacement capacity of the columns remarkably. While there are many suggestions made for the plastic hinge lengths of the columns (e.g., [5,38,39]), the plastic hinge length is assumed to be 300 and 150 mm for columns in the R35 and RW35 series, respectively (*h*/2, where h is the depth of the column). These plastic hinge lengths are also in agreement with the requirements of the Turkish Seismic Design Code [42]. Furthermore, this assumption seems to be quite realistic according to the damage distribution of columns tested during this study (Figure 5), as well as other previous studies [5,35]. Accordingly, the displacement at the top of the column was obtained by using Equation (1) through the concept of the idealized uniform distribution of plastic deformations within the assumed plastic hinge length (as also recommended by TSDC [50]). In this equation, Δ is the top displacement of the column (displacement at the actuator level); Δ_e_ is the elastic top displacement; Δ_p_ is the plastic top displacement; φ_e_ is the elastic curvature at yielding; φ is the total average curvature in the plastic hinge zone; *L*_p_ is the plastic hinge length; and *L* is the height of the column from top of foundation to the actuator level at its center. Since the examined columns constitute an isostatic structural system, the loads at any point of the load-displacement relationships are obtained by making use of the resisted moments at the critical section (mid-height of assumed plastic hinge).

(1) $$\Delta =\Delta_{e}+\Delta_{p}=\phi_{e}L^{2}/3+(\phi -\phi_{e})L_{p}(L-L_{p}/2)$$

**Figure 11:**
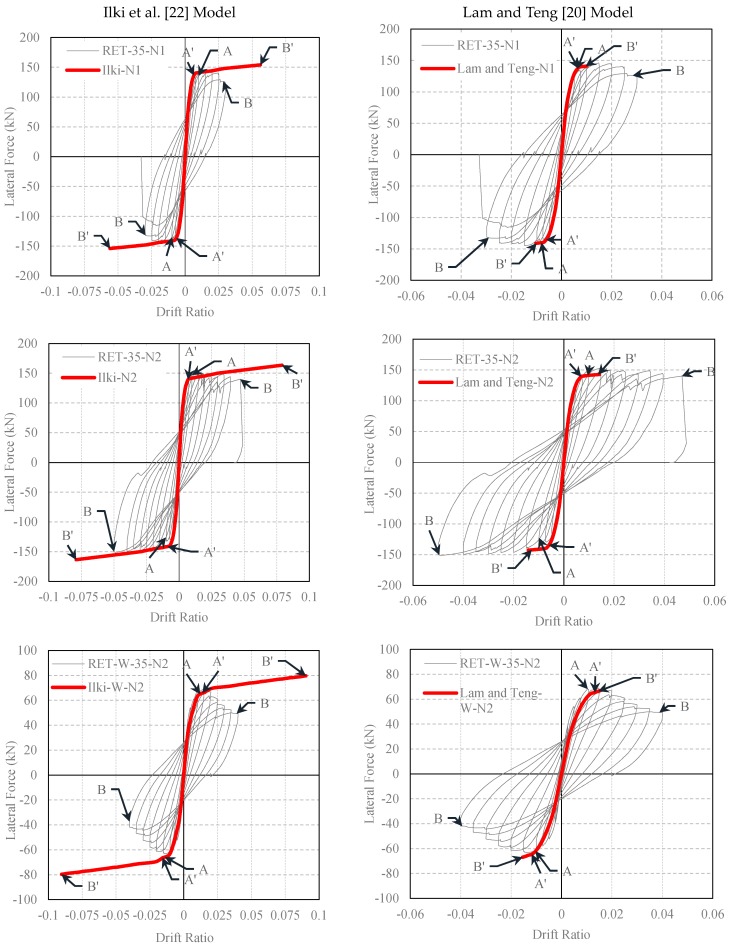
Comparison of experimental and analytical lateral load-drift relationships (confined concrete strength and strain capacity obtained by using Ilki et al. [22] and Lam and Teng [20] models).

**Figure 12:**
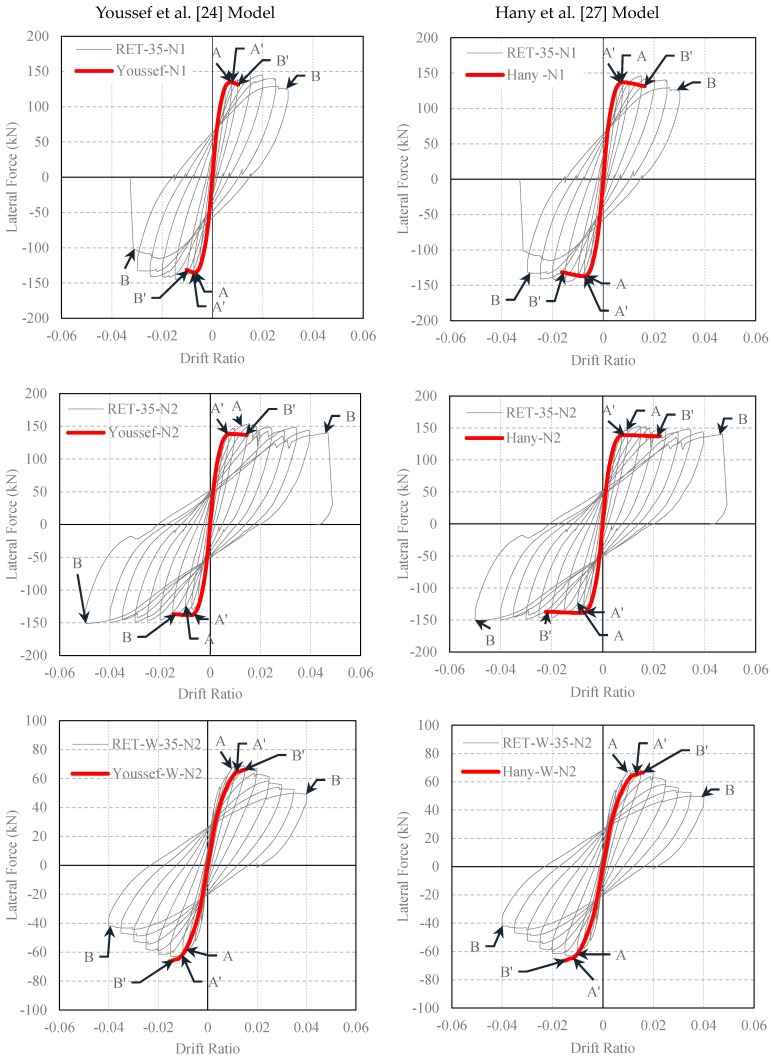
Comparison of experimental and analytical lateral load-drift relationships (confined concrete strength and strain capacity obtained by using Youssef et al. [24] and Hany et al. [27] models).

The experimental lateral load-drift ratio relationships of the tested columns and the analytical predictions made by using the confined concrete strength and ultimate strain equations of different FRP-confined concrete models are presented in Figure 11 and Figure 12. In these figures, Point A corresponds to first yielding of the longitudinal bars in tension measured during the test, whereas Point A’ is the yielding point obtained from the analytical procedure. Point B shows the drift ratio at which FRP rupture occurred during the test, while Point B’ is the corresponding point analytically determined. For better interpretation of the data for the yielding of the longitudinal reinforcement and rupture of the FRP jacket, data for A, B, A’ and B’ are tabulated in Table 8, Table 9, Table 10, Table 11 and Table 12. At first sight, for small drift ratios (i.e., drift ratios less than 0.01), all evaluated confinement models are in good agreement with the experimental results. This can also be confirmed by comparing the A and A’ points, where the first yielding of the longitudinal bars occurred. However, at higher drift ratio levels, where the behavior of FRP confined concrete has a major role, all models, except the one proposed by Ilki et al. [22], highly underestimate the drift capacities for both series (R35 and RW35). For these columns, on the other hand, the model based on the equations proposed by Ilki et al. [22] overestimates the drift capacities. Additionally, for the same model, the lateral load capacities of the tested columns are also slightly overestimated. This overestimation of lateral strength is more pronounced in the case of the column loaded in the weak direction of the cross-section (RET-W-35-N2), particularly in the post-peak region of the loading. Consequently, the models based on the strength and deformability suggestions of Lam and Teng [20], Youssef et al. [24] and Hany et al. [27] are generally too conservative in terms of ductility, whereas that of Ilki et al. [22] is non-conservative in terms of ductility and strength characteristics.

**Figure 13:**
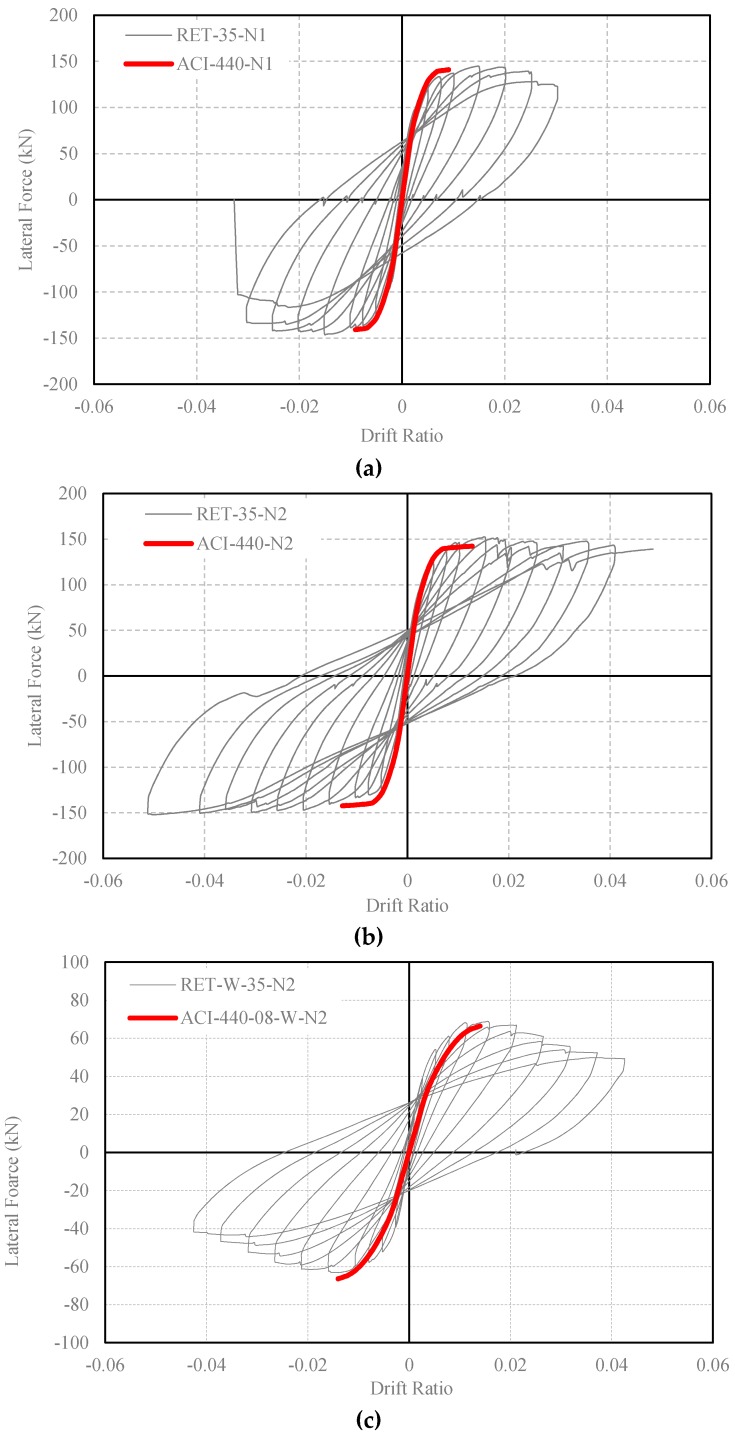
Comparison of experimental and analytical lateral load-drift relationships (confined concrete strength and strain capacity are obtained by using ACI 440.2R [48] equations): (**a**) RET-35-N1; (**b**) RET-35-N2 and (**c**) RET-W-35-N2.

Another investigation is carried out using the approach given by ACI 440.2R [48]. In this document, an FRP confinement model, which is based on the Lam and Teng model [20], is provided for increasing the ductility of reinforced concrete members. According to the ACI 440.2R [48], the FRP-confined concrete strength (*f*_cc_) and the corresponding strain (ε_cc_) can be calculated by using Equations (2) and (3). In these equations, κ_a_ and κ_b_ are the section shape factors, ε_f_ is the effective fracture strain of FRP and ψ_f_ is the safety factor. The effective fracture strain of FRP is proposed as 0.55ε_fu_. A lower limit of 0.08f_co_ is recommended for the maximum lateral confinement pressure provided by FRP confinement (*f*_l_ = 2*E*_f_*t*_f_ε_fe_*n*/D), where *E*_f_ is the tensile modulus of elasticity of FRP, *n* is the number of plies of FRP reinforcement, *t*_f_ is the nominal thickness of one ply of FRP reinforcement, ε_fe_ is the effective strain level in FRP reinforcement attained at failure and D is the diameter of the compression member of a circular cross-section. In addition, an upper limit of 0.01 is given for the ultimate axial strain of FRP-confined concrete. As seen in Table 13, where the *f*_cc_/*f*_co_, ε_cc_/ε_co_ and *f*_l_/*f*_co_ ratios are provided, it can be noticed that, for the specimen retrofitted with one ply of CFRP confinement (RET-35-N1), the *f*_l_/*f*_co_ ratio is slightly less than 0.08. In Figure 13, the experimental and analytical lateral load vs. drift ratio curves are plotted, where the equations provided by ACI 440.2R [48] were used for the ultimate strength and strain of FRP-confined concrete. From this figure, it can be seen that the ACI 440.2R [48] equations generally lead to quite conservative results in the estimation of the drift capacity for tested FRP-confined columns.

(2) $$f_{\mathrm{cc}}=f_{c}(1+\Psi_{f}3.3\kappa_{a}(f_{l}/f_{\mathrm{co}}))$$

(3) $$\varepsilon_{\mathrm{cc}}=\varepsilon_{\mathrm{co}}\left[1.50+12\kappa_{b}(f_{l}/f_{\mathrm{co}}){\left(\frac{\varepsilon_{\mathrm{fe}}}{\varepsilon_{\mathrm{co}}}\right)}^{0.45}\right]$$

**Figure 14:**
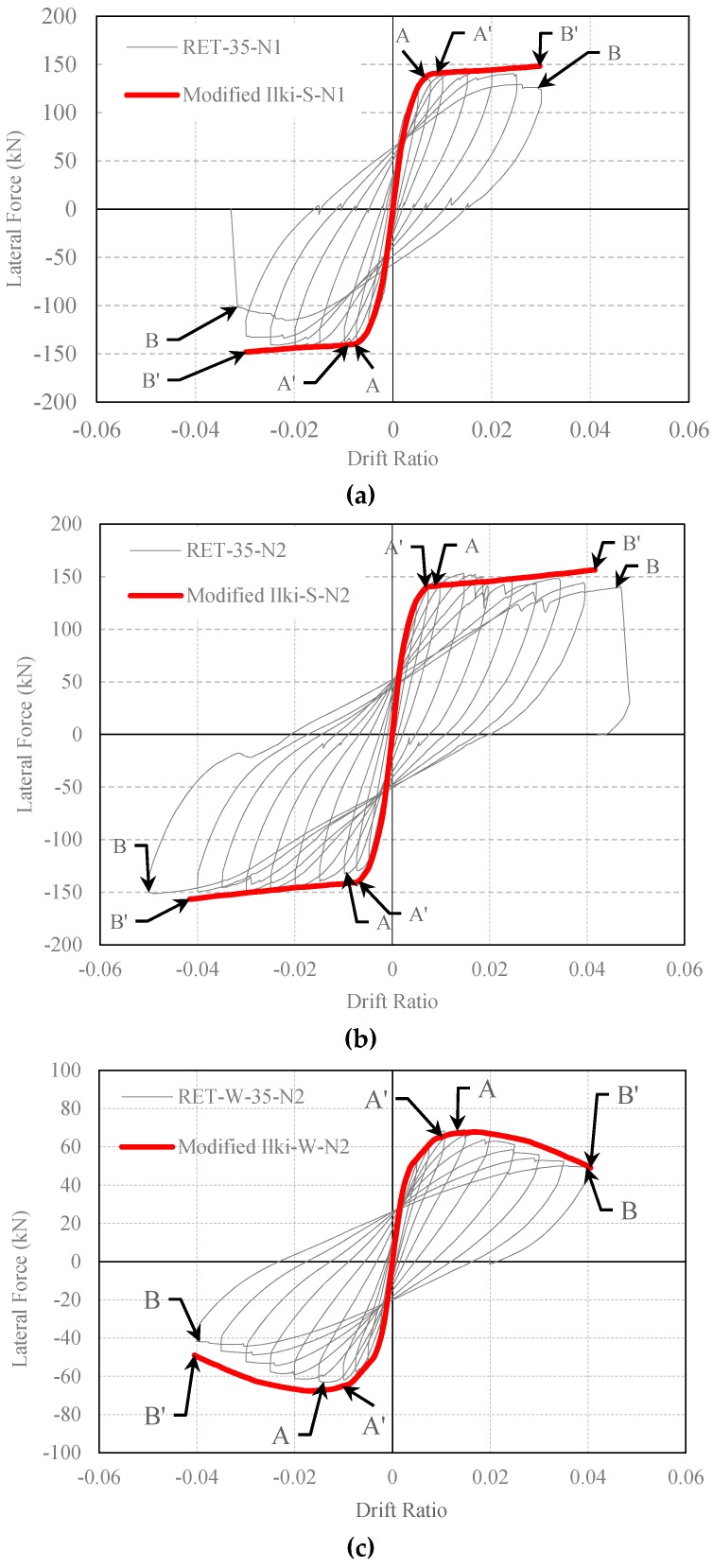
Comparison of experimental and analytical lateral load-drift relationships (confined concrete strength and strain capacity are obtained by using modified equations): (**a**) RET-35-N1; (**b**) RET-35-N2 and (**c**) RET-W-35-N2.

Although the number of tested full-scale FRP-retrofitted columns and considered ranges of test parameters are not sufficient to propose a general model for extended rectangular columns, a slight modification is suggested here on the model based on the formulations given by Ilki et al. [22] to improve the estimations of the model considering the test data. It is clear that the suggestions presented here are only valid for the tested columns or columns with similar characteristics. Unlike columns tested under concentric compression, cyclic loading was not seen to be that much effective on the increase of ultimate axial strain of FRP-confined concrete in compression. That is reasonable since, as also indicated by Ilki et al. [22], the increase in ultimate strain in case of cyclic loading of concentrically-loaded columns is related to the short-term creep of the full concrete section under high compression stresses. However, in the case of columns of real structures under seismic actions, most parts of the section are not in compression, resulting in significantly reduced effects of short-term creep. Therefore, to consider this rational change, firstly, the loading type factor LTF is omitted from the equation that estimates the ultimate strain corresponding to FRP-confined concrete strength. Secondly, as expected due to the significantly lower flexural stiffness of the FRP jacket parallel to longer direction of the columns (due to absence of cross-links, which are normally provided in the case of internal confinement), the jacket is much weaker when the column is subjected to lateral loads perpendicular to the longer side of the column (like the situation of tested column in the RW35 series). Therefore, a loading direction factor (LDF) is introduced to the equation that governs the FRP-confined concrete strength. While the value of one for LDF leads to satisfactory estimations of strength for columns tested under lateral loads perpendicular to the short side, the LDF should be considered as (*b/h*)^2^ < 1 for the lateral loading along the weak direction of the column, where b is the shorter and h is the longer dimension of the column cross-section. Accordingly, the equations given previously by Ilki et al. [22] take the form given in Equations (4) and (5). The analytical load-displacement relationships of the tested columns obtained by making use of the FRP-confined concrete axial stress-strain relationships constructed by considering the summarized modification are presented in Figure 14. As seen, the agreement of the analytical and experimental load-displacement curves is significantly better after the applied modifications.

(4) $${f^{\prime }}_{\text{cc}}=f_{\mathrm{co}}(1+2.54({f^{\prime }}_{l}/f_{\mathrm{co}}))(\mathrm{LDF})$$

(5) $$\varepsilon_{\mathrm{cc}}=\varepsilon_{\mathrm{co}}(1+(\frac{h}{b})19.27{\left({f^{\prime }}_{l}/f_{\mathrm{co}}\right)}^{0.53})$$

**Figure 15:**
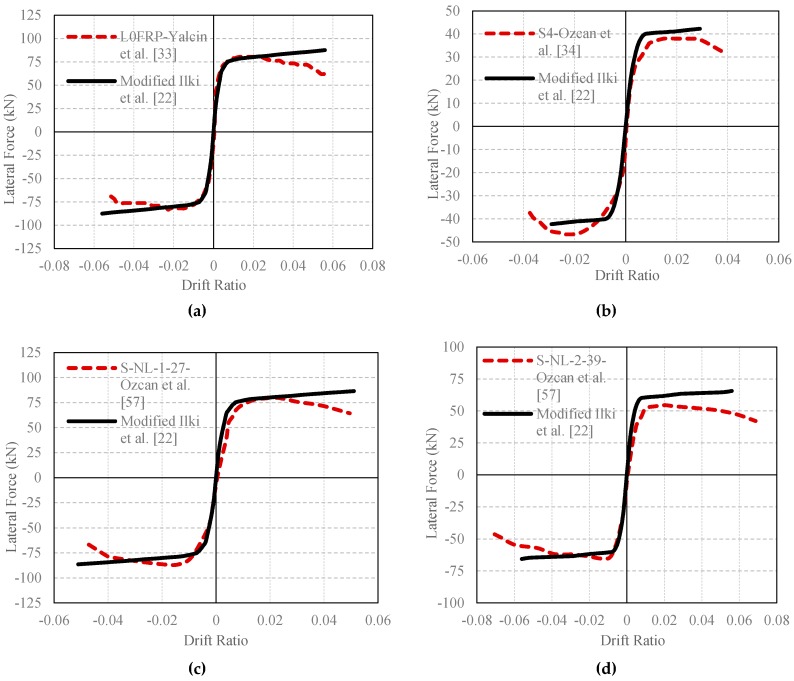
Comparison of the existing experimental results and analytical envelop relationships (modified from the Ilki et al. [22] model): (**a**) L0FRP; (**b**) S4; (**c**) S-NL-1-27 and (**d**) S-NL-2-39.

In order to verify the predictions of the modified model with column test data of other researchers, four column tests were selected from the literature [33,34,57]. The selected columns had similar characteristics with the sub-standard columns tested in this study (i.e., low quality of concrete, plain longitudinal and transverse bars and large stirrup spacing). The main specifications of the utilized columns are summarized in Table 14, and the comparisons of experimental and analytical lateral load-drift ratio envelop curves are presented in Figure 15. As seen in Figure 15, the predictions done by using the modified Ilki et al. [22] model point to a good agreement with the experimental curves obtained from the literature.

## 5. Conclusions

The conclusions derived based on experimental and analytical studies conducted on the behavior of full-scale substandard rectangular FRP-retrofitted RC columns subjected to axial force and reversed cyclic lateral loads along their strong or weak axis are summarized below.

(1) FRP retrofit of full-scale substandard rectangular RC columns with a cross-sectional aspect ratio of two successfully improved the ductility and energy dissipation capabilities.
(2) Enhancement in ductility was remarkable for columns subjected to lateral loads along their strong or weak directions (in the case of two plies of FRP jacketing approximately 90% and 50% ductility enhancement for columns loaded in the strong and weak axis, respectively).
(3) The use of different stress-strain models for FRP-confined concrete in compression led to significantly different analytical load-displacement curves for FRP-retrofitted columns. While use of the equations proposed by Ilki et al. [22] led to the overestimation of lateral load and displacement capacities, the models proposed by Lam and Teng [20], Hany et al. [27] and Youssef et al. [24] generally underestimated the ultimate displacement capacities.
(4) The model proposed by Ilki et al. [22] is modified considering the presented results of reversed cyclic lateral load tests of FRP-retrofitted columns. The modified model led to better analytical prediction of the load-displacement relationships of the tested columns. In order to verify the performance of the modified model with test data from other researchers, lateral load-drift ratio relationships of four columns are compared with the analytical curves. This verification attempt pointed to a good agreement. No doubt, further work for validation is necessary to be able to use the modified model for other cases.
(5) The use of equations provided by the ACI 440.2R [48] document resulted in conservative predictions of the lateral load and displacement capacities of columns tested along both strong and weak directions.

## Figures and Tables

**Table 1:** Mechanical properties of longitudinal and transverse reinforcement.

Reinforcing Bars	*f*_y_ (MPa)	ε_y_	*f*_max_ (MPa)	ε_max_	*f*_u_ (MPa)	ε_u_
ϕ 14	310	0.0014	452	0.19	284	0.22
ϕ 10	330	0.0015	458	0.20	285	0.23

**Table 2:** Characteristics of carbon fiber-reinforced polymer (CFRP) sheets (as indicated by the manufacturer).

Type	Thickness (mm)	Density (g/cm^3^)	Weight per Unit (g/m^2^)	Ultimate Strength (MPa)	Modulus of Elasticity (GPa)	Ultimate Strain (%)
One Dimensional CFRP	0.166	1.76	300 ± 10	4200	240	1.8

**Table 3:** Test matrix. FRP, fiber-reinforced polymer; REF, reference specimen; RET, retrofitted specimen; W, weak; N1, one layer of CFRP sheet; N2, two layers of CFRP sheets.

Specimen	Series	Loading Direction	Retrofit Method	ρ_CFRP_ (%)
REF-35	R35	Strong Direction	Reference Specimen	0
RET-35-N1	R35	Strong Direction	FRP Jacket-One Ply	0.166
RET-35-N2	R35	Strong Direction	FRP Jacket-Two Plies	0.332
REF-W-35	RW35	Weak Direction	Reference Specimen	0
RET-W-35-N2	RW35	Weak Direction	FRP Jacket-Two Plies	0.332

**Table 4:** Summary of the test results (loads are in kN).

Specimen	*P*_cr_/*DR*_cr_	*P*_y_/*DR*_y_	*P*_max_/*DR*_max_	*P*_u_/*DR*_u_	Ductility (μ = *DR*_u_/*DR*_y_)
REF-35	95.3/0.0025	134.1/0.005	136.9/0.01	115.6/0.012	2.4
RET-35-N1	97.3/0.0025	137.6/0.01	145/0.015	123.4/0.025	2.5
RET-35-N2	96.7/0.003	144.2/0.011	152.6/0.04	139.8/0.047	4.6
REF-W-35	39.4/0.003	58.2/0.01	60.7/0.015	51.2/0.02	2
RET-W-35-N2	39.8/0.003	64.5/0.011	68.9/0.025	58.5/0.03	3

**Table 5:** Concrete quality and cross-section aspect ratio ranges used for the establishment of the models and FRP efficiency ratios.

Model	*f’*_c_ (MPa)	Cross-Section Aspect Ratio *(h/b)*	*k*_ε_
Ilki et al. [22]	14.2–27.6	1, 2	0.85
Lam and Teng [20]	22.6–43.0	1, 1.5	0.586
Youssef et al. [24]	27.58–34.47	1.5	–
Hany et al. [27]	16.6–20.2	1, 1.24, 1.54	0.6

**Table 6:** Expressions for FRP-confined concrete strength and ultimate strain. LTF, loading type factor.

Model	*f*_cc_/*f*_co_	ε_cc_/ε_co_
Ilki et al. [22]	$1+2.54({f^{\prime }}_{l}/f_{\mathrm{co}})$	$1+\frac{h}{b}(\mathrm{LTF})19.27{\left({f^{\prime }}_{l}/f_{\mathrm{co}}\right)}^{0.53})$
Lam and Teng [20]	$1+3.3k_{s1}(f_{l}/f_{\mathrm{co}})$	$1.75+12k_{s2}(f_{l}/f_{\mathrm{co}}){\left(\frac{\varepsilon_{h,\mathrm{rup}}}{\varepsilon_{\mathrm{co}}}\right)}^{0.45}$
Youssef et al. [24]	$0.5+1.225{\left({f^{\prime }}_{l}/{f^{\prime }}_{c}\right)}^{\frac{3}{5}}$	$0.004325+0.02625({f^{\prime }}_{l}/{f^{\prime }}_{c}){\left(\frac{f_{\mathrm{ju}}}{E_{j}}\right)}^{\frac{1}{5}}$
Hany et al. [27]	$0.7+4.62(\frac{A_{e}}{A_{c}}){\left(\frac{b}{h}\right)}^{0.92}(f_{l}/f_{\mathrm{co}})$	$3.89+14.76(\frac{A_{e}}{A_{c}}){\left(\frac{h}{b}\right)}^{0.94}(f_{l}/f_{\mathrm{co}})$

**Table 7:** Main parameters calculated for the investigated models.

Model	*f’_l_* (*N* = 1) (MPa)	*f’_l_* (*N* = 2) (MPa)	*f*_cc_*/f*_co_ (*N* = 1)	*f*_cc_*/f*_co_ (*N* = 2)	ε_cc_/ε_co_ (*N* = 1)	ε_cc_/ε_co_ (*N* = 2)
Ilki et al. [22]	1.1	2.15	1.17	1.34	10.22	14.31
Lam and Teng [20]	1.25	2.51	1.03	1.07	3.18	4.62
Youssef et al. [24]	1.34	2.68	0.81	0.91	5.1	6.31
Hany et al. [27]	1.2	2.44	0.76	0.9	3.49	4.81

**Table 8:** Yielding and ultimate load-drift ratio data for the experimental and analytical results (Ilki et al. [22]).

Specimen	A: Experimental Yielding Point		A’: Analytical Yielding Point		B: Experimental Ultimate Point		B’: Analytical Ultimate Point	
	Load (kN)	Drift Ratio	Load (kN)	Drift Ratio	Load (kN)	Drift Ratio	Load (kN)	Drift Ratio
(RET-35-N1)	137.6	0.01	140.8	0.01	132.2	0.03	154	0.056
(RET-35-N2)	144.2	0.011	143.2	0.01	150.3	0.05	163.5	0.079
(RET-W-35-N2)	64.5	0.011	67.8	0.01	49.3	0.04	79.6	0.09

**Table 9:** Yielding and ultimate load-drift ratio data for the experimental and analytical results (Lam and Teng [20]).

Specimen	A: Experimental Yielding Point		A’: Analytical Yielding Point		B: Experimental Ultimate Point		B’: Analytical Ultimate Point	
	Load (kN)	Drift Ratio	Load (kN)	Drift Ratio	Load (kN)	Drift Ratio	Load (kN)	Drift Ratio
(RET-35-N1)	137.6	0.01	139.3	0.007	132.2	0.03	141.8	0.011
(RET-35-N2)	144.2	0.011	140.1	0.007	150.3	0.05	142.5	0.015
(RET-W-35-N2)	64.5	0.011	65.3	0.01	49.3	0.04	66.9	0.016

**Table 10:** Yielding and ultimate load-drift ratio data for the experimental and analytical results (Youssef et al. [24]).

Specimen	A: Experimental Yielding Point		A’: Analytical Yielding Point		B: Experimental Ultimate Point		B’: Analytical Ultimate Point	
	Load (kN)	Drift Ratio	Load (kN)	Drift Ratio	Load (kN)	Drift Ratio	Load (kN)	Drift Ratio
(RET-35-N1)	137.6	0.01	135.4	0.007	132.2	0.03	131.1	0.011
(RET-35-N2)	144.2	0.011	138.4	0.007	150.3	0.05	136.6	0.015
(RET-W-35-N2)	64.5	0.011	65.3	0.01	49.3	0.04	66.7	0.015

**Table 11:** Yielding and ultimate load-drift ratio data for the experimental and analytical results (Hany et al. [27]).

Specimen	A: Experimental Yielding Point		A’: Analytical Yielding Point		B: Experimental Ultimate Point		B’: Analytical Ultimate Point	
	Load (kN)	Drift Ratio	Load (kN)	Drift Ratio	Load (kN)	Drift Ratio	Load (kN)	Drift Ratio
(RET-35-N1)	137.6	0.01	137.2	0.007	132.2	0.03	131.5	0.017
(RET-35-N2)	144.2	0.011	137.8	0.007	150.3	0.05	137.2	0.023
(RET-W-35-N2)	64.5	0.011	64.5	0.01	49.3	0.04	66.7	0.016

**Table 12:** Yielding and ultimate load-drift ratio data for the experimental and analytical results (modified from Ilki et al. [22]).

Specimen	A: Experimental Yielding Point		A’: Analytical Yielding Point		B: Experimental Ultimate Point		B’: Analytical Ultimate Point	
	Load (kN)	Drift Ratio	Load (kN)	Drift Ratio	Load (kN)	Drift Ratio	Load (kN)	Drift Ratio
(RET-35-N1)	137.6	0.01	140.5	0.01	132.2	0.03	148.2	0.03
(RET-35-N2)	144.2	0.011	142.8	0.01	150.3	0.05	155.6	0.042
(RET-W-35-N2)	64.5	0.011	66.5	0.013	49.3	0.04	49.04	0.04

**Table 13:** Predictions of the American Concrete Institute (ACI) 440.2R [48] model for the tested specimens.

Specimen	Series	*f*_cc_/*f*_co_	ε_cc_/ε_co_	*f*_l_/*f*_co_
RET-35-N1	R35	1.04	1.7	0.078
RET-35-N2	R35	1.09	2.2	0.155
RET-W-35-N2	RW35	1.09	2.2	0.155

**Table 14:** The specifications of selected experimental specimens from the literature.

Specimen	Cross Section (mm × mm)	*f’*_c_ (MPa)	Number of FRP Plies	Axial Load Level (%)	Longitudinal Reinforcement	Stirrup
L0FRP [33]	200 × 400	16	3	27	6 ϕ14	ϕ8@200 mm
S4 [34]	200 × 400	9	1	35	8 ϕ18	ϕ10@200 mm
S-NL-1-27 [57]	350 × 350	19.4	1	27	8 ϕ18	ϕ10@200 mm
S-NL-2-39 [57]	350 × 350	11.4	2	39	8 ϕ18	ϕ10@200 mm

## Abbreviations

The following abbreviations are used in this manuscript:

ACI	American Concrete Institute
CFRP	Carbon fiber reinforced polymer
FRP	Fiber reinforced polymer
LVDT	Linear variable differential transformer
LTF	Loading type factor
LDF	Loading direction factor
RC	Reinforced concrete
TSDC	Turkish seismic design code
